# Warming effects on photosynthesis of subtropical tree species: a translocation experiment along an altitudinal gradient

**DOI:** 10.1038/srep24895

**Published:** 2016-04-22

**Authors:** Yiyong Li, Juxiu Liu, Guoyi Zhou, Wenjuan Huang, Honglang Duan

**Affiliations:** 1 Key Laboratory of Vegetation Restoration and Management of Degraded Ecosystems, South China Botanical Garden, Chinese Academy of Sciences, Guangzhou 510650, China; 2 Graduate University of Chinese Academy of Sciences, Beijing 100049, China; 3 Institute of Ecology and Environmental Science, Nanchang Institute of Technology, Nanchang, Jiangxi 330099, China

## Abstract

Ongoing climate warming induced by human activities may have great impacts on trees, yet it remains unresolved how subtropical tree species respond to rising temperature in the field. Here, we used downward translocation to investigate the effects of climate warming on leaf photosynthesis of six common tree species in subtropical China. During the experimental period between 2012 and 2014, the mean average photosynthetic rates (*A*_sat_) under saturating light for *Schima superba*, *Machilus breviflora*, *Pinus massoniana* and *Ardisia lindleyana* in the warm site were7%, 19%, 20% and 29% higher than those in the control site. In contrast, seasonal *A*_sat_ for *Castanopsis hystrix* in the warm site were lower compared to the control site. Changes in *A*_sat_ in response to translocation were mainly associated with those in leaf stomatal conductance (*g*_s_) and photosynthetic capacity (RuBP carboxylation, RuBP regeneration capacity). Our results imply that climate warming could have potential impacts on species composition and community structure in subtropical forests.

Tropical and subtropical forests can provide crucial ecosystem services to natural systems and human kind (i.e., carbon sequestration^1^,^2^, biodiversity conservation, climatic regulation). As a result of climate change, global mean temperatures are projected to increase 1–5 °C in the next 50–100 years^3^. Such warming may have deleterious consequences on subtropical and tropical forests since many tree species occur near the thermal optimum^4^. Over the last decade, field observational studies across subtropical and tropical forests have documented changes in forest structure and tree mortality which have been attributed to climate warming and severe droughts^5^,^6^,^7^,^8^. However, the physiological mechanisms that govern such shifts are not well understood. It is well established that one of the primary physiological targets of warming is photosynthesis^9^,^10^ which acts as the fundamental basis for carbon accumulation, growth and biomass production of plants. Therefore, an improved understanding of the photosynthetic mechanisms underpinning plant response to climate warming in tropical and subtropical species may provide insights into shifts in forest community composition under future climates.

Photosynthesis is strongly affected by temperature. Photosynthesis usually increases as temperature rises when below the thermal optimum, while it may decline once the temperature exceeds the thermal optimum^11^. The effects of temperature on photosynthesis can be divided into two primary driving mechanisms: stomatal effects (indirect) and biochemical effects (direct)^12^. The increased leaf-to-air vapour pressure (VPD) associated with rising temperature has long been recognized as a major factor determining stomatal responses, thereby affecting plant carbon assimilation under warming^12^,^13^. Additionally, biochemical processes are also temperature-dependent. Responses of biochemical processes in the mesophyll are characterized by the modulation of the rates of activity of photosynthetic enzymes and the electron transport chain^14^. The maximum rate of Rubisco (the main carboxylating enzyme of photosynthesis) carboxylation (*V*_cmax_) and the maximum rate of photosynthetic electron transport (*J*_max_)^9^, are associated with the responses of photosynthesis^15^,^16^.

Over recent decades, results from photosynthesis research among warming experiments are inconclusive: elevated temperature has been found to increases^17^,^18^,^19^, decreases^20^,^21^,^22^,^23^ or no impacts^24^,^25^,^26^,^27^ on *A*_sat_. While the photosynthetic responses of temperate plant species under climate warming are comparatively well studied^17^,^28^, the responses of tropical tree species remain unexplored. Previous studies have found that species exposed to greater seasonal variation in temperature show greater in temperate optima. However, Cunningham and Read *et al.*^29^ compared four temperate and four tropical species, and observed greater capacities for tropical species to acclimate higher temperature compared to temperate species. These inconsistent results require better understandings in to what degree tropical and subtropical species respond to increasing temperature^12^,^30^.

Inferring how climate change affects plants has been typically relied on one of three approaches: experiments with warming facilities, historical comparisons, and space-for-time substitutions derived from sampling along environmental gradients^31^. Researchers who have investigated temperature effects have often studied seedlings in growth plots kept at constant temperatures^32^,^33^ and less is known about thermal responses in natural settings with temperature variability. Manipulative studies in the field remain a logistical challenge for global change scientists^34^,^35^. To measure species across a wide range of temperatures in natural systems, field studies have made use of latitudinal and altitudinal gradients, as well as seasonal changes in temperature^36^,^37^. While past research using growth chambers and temperature gradients has detected acclimation across wide temperature ranges, it is uncertain whether acclimation will also occur when plants are subjected to smaller temperature increases (1–2 °C).

Here, we conducted a transplant experiment in two sites along an altitudinal gradient. Using this approach, we assessed the effects of temperature on photosynthetic performance of co-existing species in subtropical China. We selected 6 subtropical tree species that are from different functional types (i.e., tree vs shrub, broadleaved vs coniferous tree) to determine photosynthetic responses to rising temperature. Our specific objectives are to address the following questions: 1) how does climate warming affect photosynthesis of different plant species in subtropical China? 2) What biochemical and stomatal mechanisms are involved in the changes in photosynthesis?

## Results

### Environmental variables

**Figure 1:**
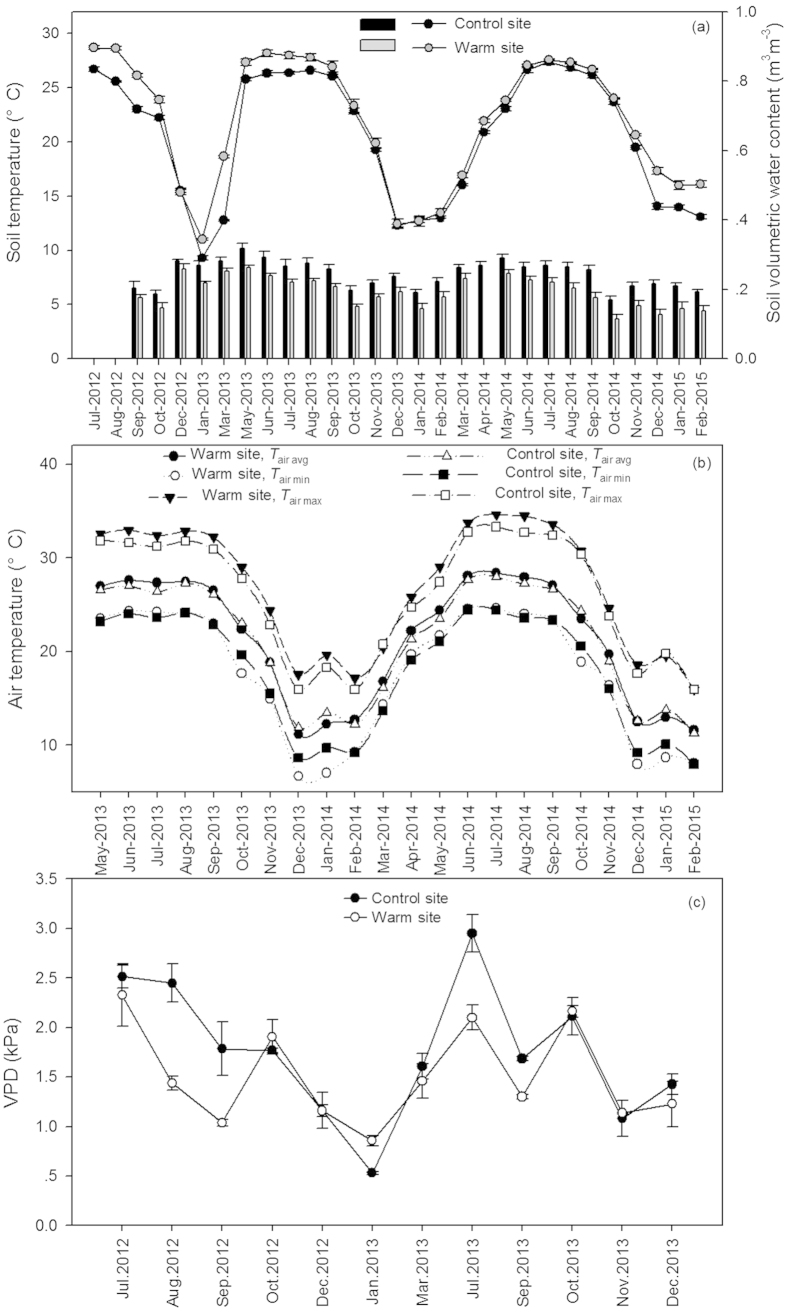
Monthly mean soil temperature (lines) and volumetric soil water content (bars) (**a**) monthly maximum, minimum and mean air temperature (**b**) monthly leaf-to-air vapor pressure deficit (**c**) VPD) at the warm and control site.

The monthly air and soil temperatures at the warm site were distinctly higher than those at the control site. Mean soil temperature was 0.93 °C higher in the warm site than in the control site (Fig. 1a, *p* < 0.01). Soil moisture and vapor pressure deficit (VPD) were significantly lower in the warm site than in the control site (Fig. 1a,c, *p* < 0.01). During the experimental period, mean of average, maximum and minimum monthly air temperature were 0.90 °C, 0.95 °C, and 1.29 °C higher in the warm site than those in the control site, respectively (Fig. 1b, *p* < 0.01).

### Responses of *A*_sat_

**Figure 2:**
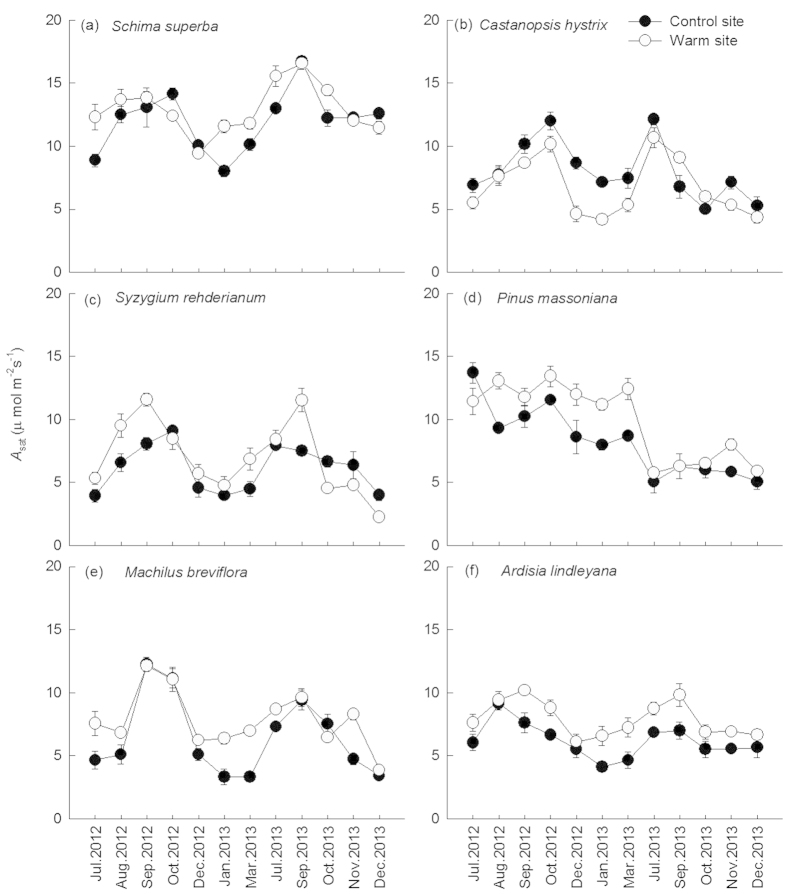
Seasonal dynamics of photosynthetic rate (*A*_sat_) in *Schima superba* (**a**), *Castanopsis hystrix* (**b**), *Syzygium rehderianum* (**c**)*, Pinus massoniana* (**d**), *Machilus breviflora* (**e**) and *Ardisia lindleyana* (**f**) grown in the warm and control site. Error bars are standard error (n = 3).

Over the entire experimental period, *A*_sat_ of the six tree species showed similar seasonal patterns (Fig. 2), with high *A*_sat_ occurred during the summer and low *A*_sat_ occurred during the winter. Downward translocation significantly increased annual mean average *A*_sat_ of *S. superba* (+7%), *P. massoniana* (+20%), *M. breviflora* (+19%), *A. lindleyana* (+29%) (*p* < 0.05 for all) (Tables 1 and 2); however, it decreased that of *C*. *hystrix* (−10%) (*p* < 0.05, Tables 1 and 2) and had no significant effect on *S. rehderianum*. The impact of measuring date on *A*_sat_ of all species was statistically significant (*p* < 0.05, Table 1), with greater *A*_sat_ in the warm-wet seasons and lower rates in the cool-dry ones (Table 2). Translocation treatment significantly increased mean *A*_sat_ for *S. superba* and *S. rehderianum* in wet-warm season (12% and 36%, respectively) but decreased it in cold-dry season (3% and 4%, respectively, not significant). By contrast, the magnitude of effects in *A*_sat_ for *P. massoniana* and *C. hystrix* was greater in cool-dry season (29% and −13%, respectively) compared to warm-wet season (8% and −8%, respectively) (Table 2).

**Figure 3:**
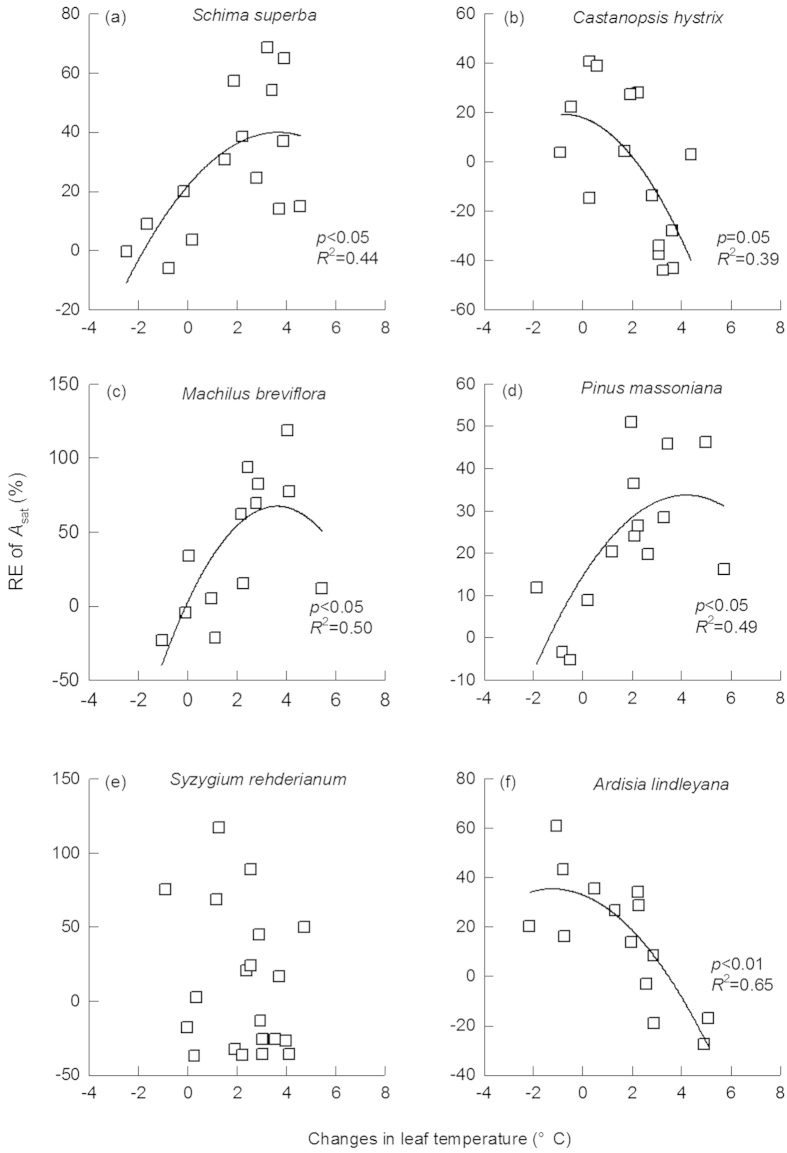
Correlations between relative effect (RE) of photosynthetic rate (*A*_sat_) with changes in leaf temperature. Data were fitted using a nonlinear regression (solid line). The adjusted *R*^2^ value and its significance for each fitting are shown.

**Figure 4:**
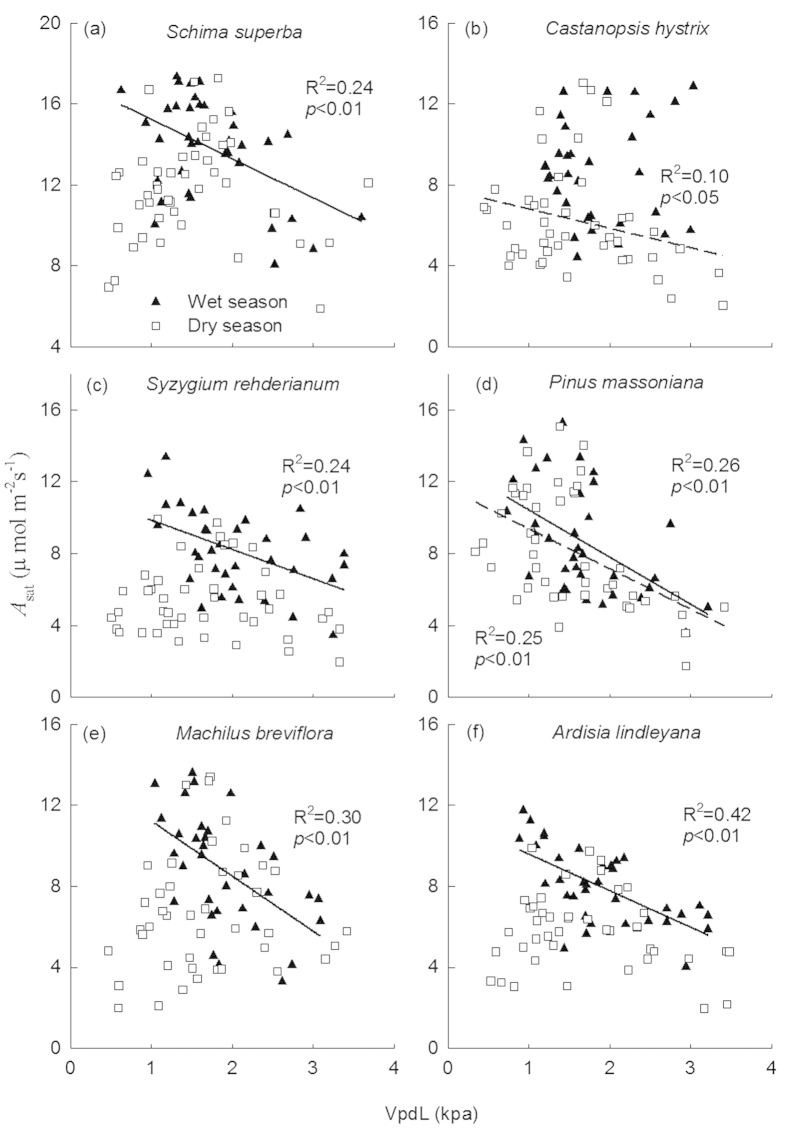
Correlations between photosynthetic rate (*A*_sat_) with vapor pressure deficit (VpdL) in wet season (solid line) and dry season (dashed line), respectively. The adjusted *R*^2^ value and its significance for each fitting are shown.

The RE of *A*_sat_ was positively correlated with the increases in leaf temperature for *S. superba* (Fig. 3a, *R*^2^ = 0.44), *M. breviflora* (Fig. 3c, *R*^2^ = 0.50), *P. massoniana* (Fig. 3d, *R*^2^ = 0.49), but negatively correlated with that for *C. hystrix* (Fig. 3b, *R*^2^ = 0.39)and *A. lindleyana* (Fig. 3f, *R*^2^ = 0.65). *A*_sat_ were negatively correlated with VpdL in wet season for all species except for *C. hystrix* (Fig. 4, *p* < 0.01 for all). The correlations between *A*_sat_ and VpdL in dry season were significant for *C. hystrix*, *P. massoniana*, and *A. lindleyana*. (Fig. 4, *p* < 0.05)

### Responses of *g*_s_

**Figure 5:**
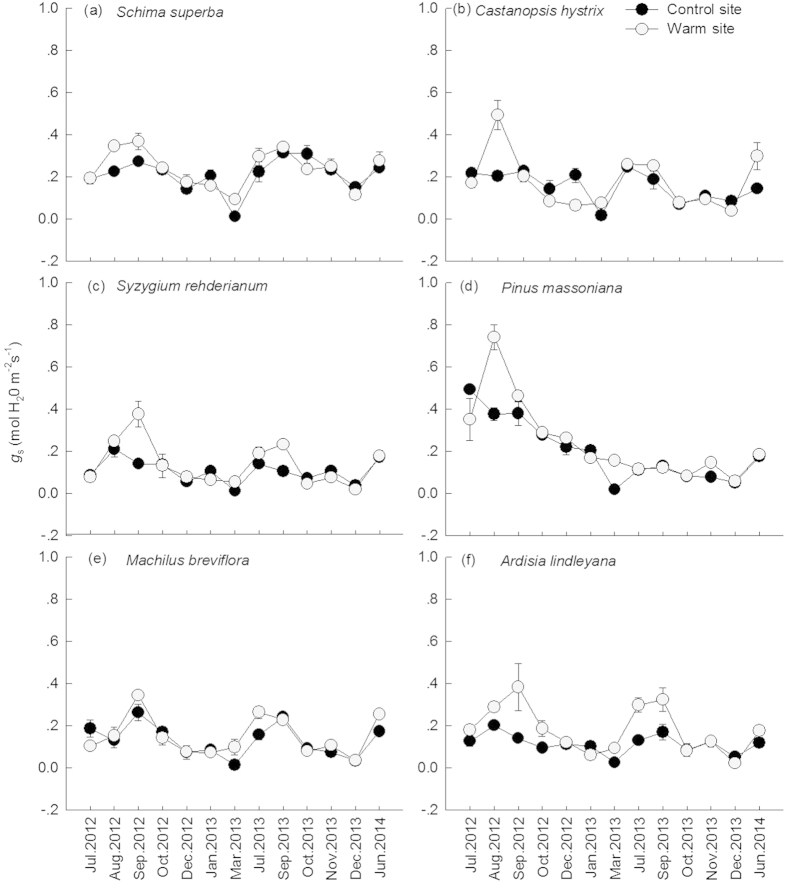
Seasonal dynamics of stomatal conductance (*g*_s_) in *Schima superba* (**a**), *Castanopsis hystrix* (**b**), *Syzygium rehderianum* (**c**)*, Pinus massoniana* (**d**), *Machilus breviflora* (**e**) and *Ardisia lindleyana* (**f**) grown in the warm site and control site. Error bars are standard error (n = 3).

**Figure 6:**
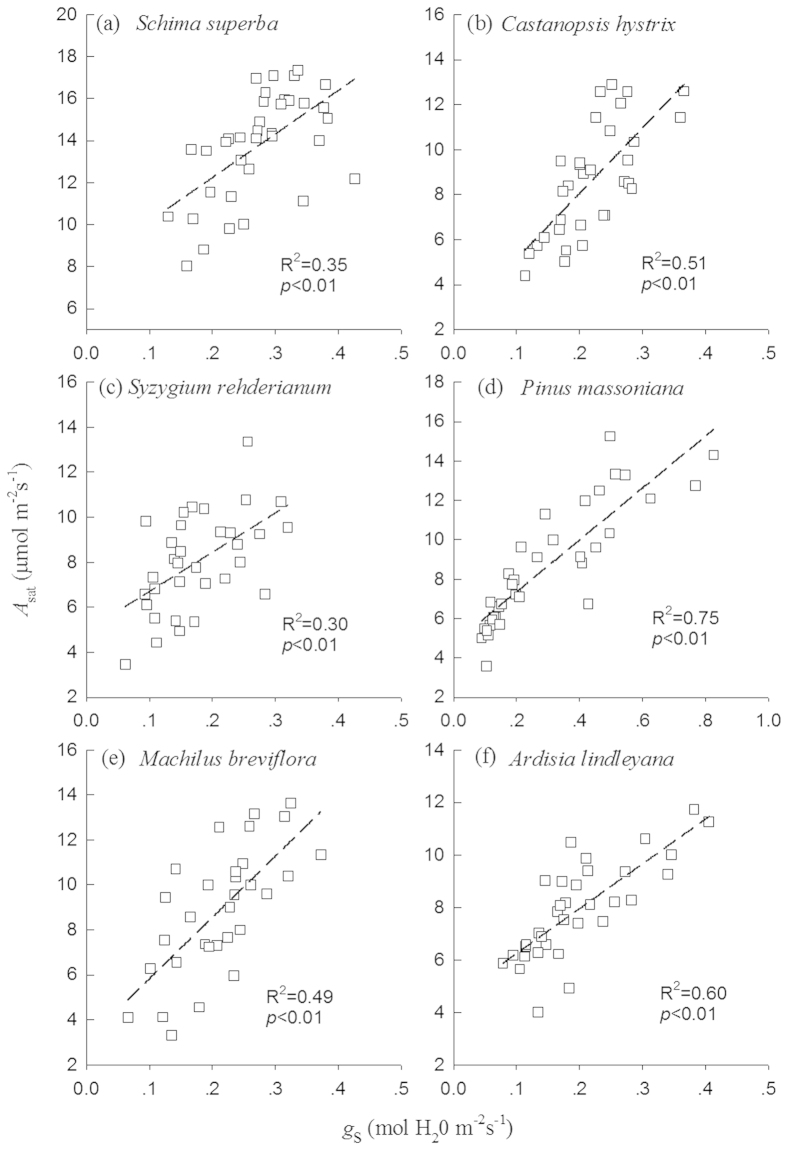
Correlations between photosynthetic rate (*A*_sat_) and stomatal conductance (*g*_s_) in *Schima superba, Castanopsis hystrix, Syzygium rehderianum, Pinus massoniana, Machilus breviflora and Ardisia lindleyana*.

**Figure 7:**
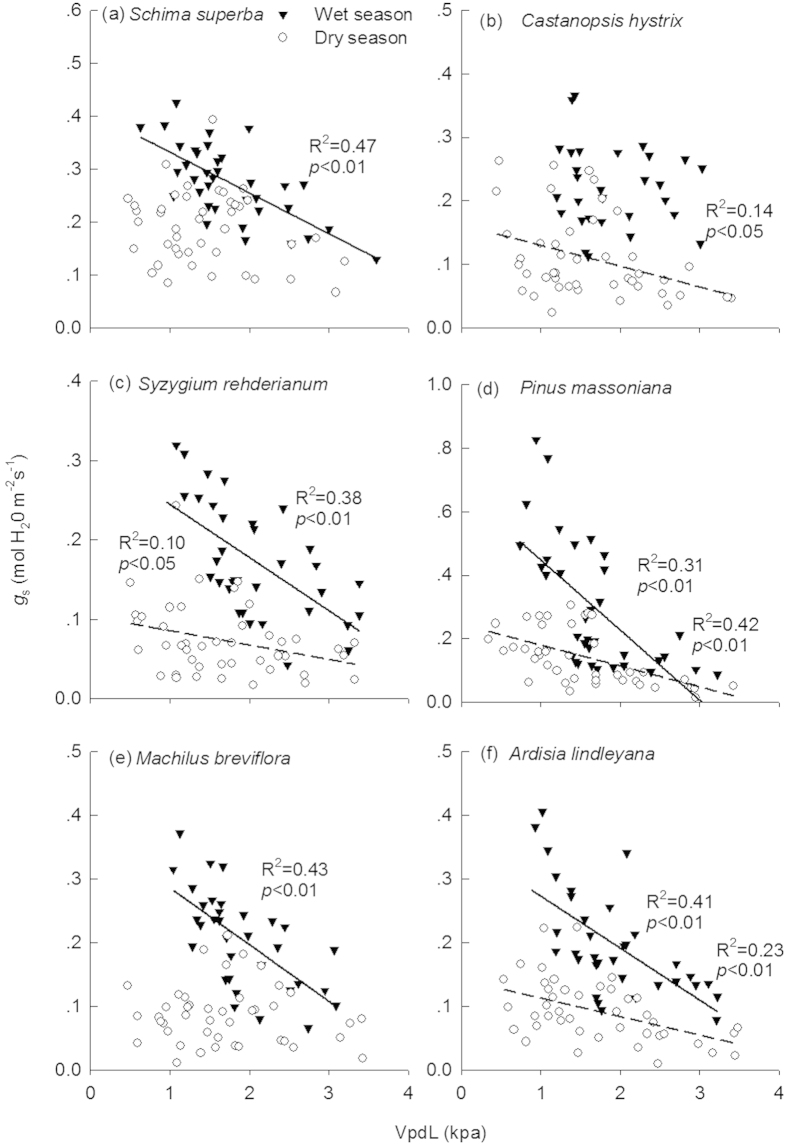
Correlations between stomatal conductance (g_s_) with vapor pressure deficit (VpdL) in wet season (solid line) and dry season (dashed line), respectively. The adjusted R^2^ value and its significance for each fitting are shown.

Consistent with *A*_sat_, leaf *g*_s_ of the six species showed similar seasonal patterns (Fig. 5). Translocation significantly increased *g*_s_ of *S. rehderianum*, *P. massoniana*, and *A. lindleyana* (*p* < 0.01 for all) (Tables 1 and 2). For all tree species, there were significant relationship of *A*_sat_ with *g*_s_ in our study (Fig. 6, *p* < 0.01 for all). *g*_s_ were negatively correlated with VpdL in wet season for all species except for *C. hystrix* (Fig. 7, *p* < 0.01 for all). The correlations between *g*_s_ and VpdL in dry season were significantly for *C. hystrix*, *S. rehderianum*, *P. massoniana*, and *A. lindleyana*. (Fig. 7, *p* < 0.05)

### Responses of *V*_cmax_, *J*_max_ and TPU

**Figure 8:**
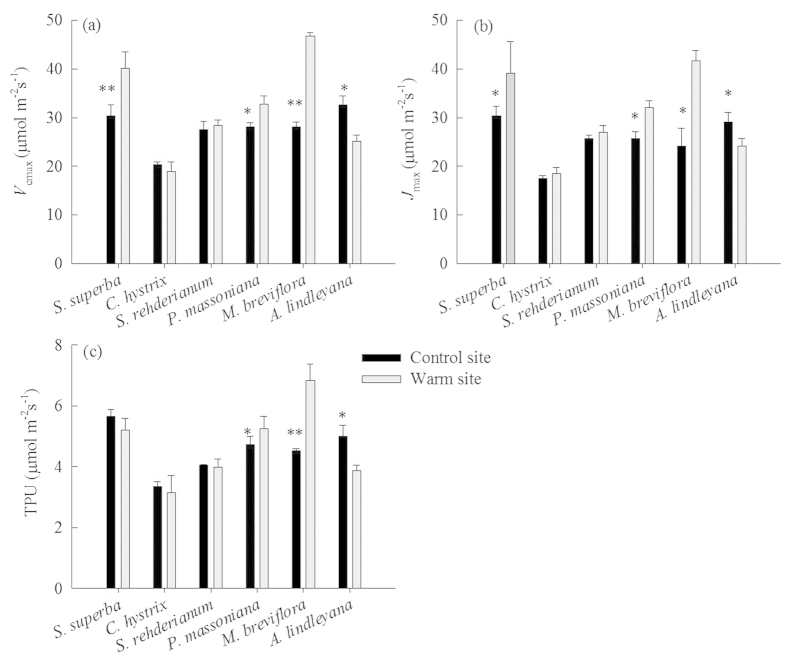
The maximum rate of maximum rate of photosynthetic carboxylation (**a**, *V*_cmax_), the maximum rate of photosynthetic electron transport (**b**, *J*_max_) and triose-phosphate utilization (**c**, TPU) for *Schima superba*, *Castanopsis hystrix*, *Syzygium rehderianum, Pinus massoniana*, *Machilus breviflora* and *Ardisia lindleyana* grown in the warm site and control site. Error bars are standard error (n = 3). Asterisks (*) and (**) indicate that there are significant differences at *p* < 0.05 and *p* < 0.01 between the warm site and the controls, respectively.

The six species showed different responses of photosynthetic capacity to translocation (Fig. 8). Downward translocation significantly increased *V*_cmax_, *J*_max_ and TPU of *S. superba*, *P. massoniana* and *M. breviflora* (*p* < 0.05); however, it significantly decreased those of *A. lindleyana* (*p* < 0.05) and had no significant effect on *C*. *hystrix* and *S. rehderianum* (*p* > 0.05).

## Discussion

Our experimental set-up simulated climate warming by translocation of plant-soils to lower elevations. In our experiment, soil temperature and soil moisture at the lower elevation were significantly different from those in control site, resulting in a 0.90 °C and 0.93 °C increase in mean air and soil temperature, respectively (Fig. 1). Natural geographical gradients provide an excellent natural laboratory to investigate the potential effects of climate warming on terrestrial organisms^38^. However, different plant ecotypes formed in their own habitats increase the difficulty of comparison and analysis. To overcome these problems accompanied by geographical gradients, we conduct transplant experiments to allow comparisons within the same ecotype. We are rarely aware of this combination application to studies specifically regarding forests, although this approach has been conducted in grass or shrub^39^,^40^. Therefore, altitudinal gradients are still an underutilized resource in this respect. Although co-occurring changes in other environmental factors might counteract the effect of elevated temperature on photosynthesis, moving on from these geographical effects to clearly inferring a temperature signal appears as an important step.

The overall objective of this study was to improve the limited understanding of temperature responses of photosynthesis in subtropical tree species. The effects of warming on tree or seedling photosynthesis have been previously studied, but the results have shown varying patterns^17^,^21^,^25^. Furthermore, whether or not tropical and subtropical species are operating near thermal thresholds remained unclear. For example, Vårhammar *et al.*^21^ found negative effects of elevated temperature on tropical African trees. Several leaf and canopy measurements confirmed that gas exchange decreased with increased temperature^41^. Whereas to date, few temperature response assessments have focused on subtropical trees. In our study, we found significant greater *A*_sat_ in *S.superba*, *P. massoniana*, *M. breviflora* and *A. lindleyana* grown in warm site (Tables 1 and 2), with positive relationships between photosynthesis and changes in leaf temperature (Fig. 6). This finding was rejected to the hypothesis that subtropical tree species may be near a high temperature threshold^42^,^43^. In contrast to those species, *C*. *hystrix* was found to have lower *A*_sat_ grown in warm site (Tables 1 and 2), indicating that the photosynthesis of these subtropical tree species have species-specific responses to rising temperatures. It is also consistent with findings of previous studies on temperate and boreal species^44^,^45^,^46^,^47^. Seasonal responses of *A*_sat_ to warming for the two species (*S. superba* and *S. rehderianum*) were similar, but were different from other two species (*P. massoniana* and *C. hystrix*).

The response of photosynthesis under elevated temperature was usually associated with mesophyll (direct) effects or stomatal (indirect) effects^12^. According to the models of Farquhar *et al.*^9^, the balance between *V*_cmax_ and *J*_max_ of ribulose-1,5-bisphosphate (RuBP) determines the temperature dependence of photosynthesis^48^. In our study, *S. superba*, *P. massoniana* and *M. breviflora* which had higher *A*_sat_ at warm site also experienced increases in *J*_max_ and *V*_cmax_ (Fig. 8), indicating greater photosynthetic electron transport and Rubisco carboxylation^9^. This finding conforms to the models of Farquhar *et al.*^9^, and it is also consistent with previous experimental evidences^49^,^50^. Leading models propose that the inhibition of *A*_sat_ at elevated temperature is a function of either declining capacity of RuBP activation, or Rubisco activase to maintain Rubisco in an active configuration^51^,^52^. However, in our study, *A*_sat_ was decreased for *C. hystrix* in the warm site, yet the decreases of photosynthetic capacity were not statistically significant. For *A. lindleyana*, we found an enhancement in *A*_sat_ but a decrease in photosynthetic capacity (Fig. 8). This differs from previous studies in that *V*_cmax_ or *J*_max_ changed, inferring a shift in resource investment away from Rubisco. Bernacchi *et al.*^53^ also found increased *A*_sat_ even though there was significant decrease in *V*_cmax_. This may result from changes in mesophyll conductance (*g*_m_), the transfer conductance of CO_2_ from the intercellular air-space to the site of carboxylation within the chloroplast, has been shown to be a significant limitation to photosynthesis^54^. In addition, *C. hystrix* had lower optimum temperatures for photosynthesis compared with warm-adapted species.

Stomatal regulation of the internal CO_2_ concentration is well known for determining the temperature response of photosynthesis^55^,^56^,^57^. In our experiment, enhancement or reduction of *A*_sat_ due to downward translocation were largely associated with the changes in *g*_s_ in all six species (Fig. 4), proposing *g*_s_ as a screening tool for gas exchange efficiency^44^,^58^. The leaf to air vapour pressure difference (VPD) is the directly driving force of *g*_s_^59^. Temperature and VPD are often merged together as a combined effect because the strong correlation between the two parameters^60^,^61^. *g*_s_ is negatively correlated with VPD, which is supported by many previous studies^62^,^63^ as well as our experiment. VPD depends on the magnitude of changes in air temperature and humidity. VPD often increases as temperature rises if relative humidity remains constant or decreases^60^,^61^. However, if relative humidity increases to a greater extent than temperature, VPD can exhibit declines. In our study, VPD was found lower in the warm site when compared to the control site (Fig. 1c), mainly due to the larger air relative humidity in the warm site. Therefore, lower VPD at the warm site can contribute to the increases in *g*_s_ and *A*_sat_.

The inter-specific variation in responses of photosynthesis to elevated temperature also highlights the importance of considering leaf and life-history traits (i.e. succession stage, morphology traits, leaf nutrients) influencing the leaf energy balance when evaluating plant sensitivity to air temperature. For example, Cheesman *et al.*^64^ have demonstrated negative effect of warming on growth in seedlings of tropical climax species than in those of pioneer species. Consistent with this, in our study, *C. hystrix* is one of the most important and dominant climax species of the evergreen forests in subtropical China^65^, photosynthesis of which was negatively affected by warming. The response of photosynthesis to warming might also be related to different leaf morphology traits. Higher specific leaf area associated with efficient light capture could have led to larger assimilation gains^66^. Recently, in connection with the studies of *g*_s_ under climate warming, response of stomatal anatomy and density are of special interest^67^,^68^. Stomatal trait has a potential to set the limit for maximum *g*_s_ for gas diffusion^69^. In the long term, changes in *g*_s_ can also be influenced by stomatal density, stomatal index and stomatal aperture^70^. These results indicate that inter-specific variation in photosynthesis is often controlled by differences in leaf traits. More research is needed to better understand the possible tradeoffs of different strategies.

In conclusion, the results demonstrated that translocation differentially affected photosynthesis of the dominant subtropical species and photosynthetic responses to translocation were season-dependent. The seasonal mean *A*_sat_ of *Schima superba*, *Machilus breviflora*, *Pinus massoniana* and *Ardisia lindleyana* in the warm site were significantly greater than those in the control site, but it was not affected for *Syzygium rehderianum* and declined for *Castanopsis hystrix*. The responses of *A*_sat_ were largely determined by *g*_s_, which was sensitive to environmental variables (such as VPD). Amongst the six tree species, *Schima superba*, *Ardisia lindleyana*, and *Machilus breviflora* were more sensitive species to translocation. The specie-specific and season-dependent photosynthetic responses under translocation indicate that climatic warming could have potential impacts on species composition and community structure in subtropical forests in China.

## Materials and Methods

### Study site

The present study was conducted at the Dinghushan Biosphere Reserve, located in the central Guangdong Province in southern China (112°10′E, 23°10′N). This reserve lies between 10 and 1000 m above sea level (a.s.l.). This region is characterized by a typical south subtropical monsoon climate, with a mean annual temperature of 21 °C with the maximum and minimum monthly mean temperature being 28.0 °C in July and 12.6 °C in January, respectively. The mean annual precipitation of 1956 mm, of which nearly 80% falls in the warm-wet season (April-September) and 20% in the cold-dry season (October-March).

### Experimental design

We conducted a common garden translocation experiment at two sites (300 m and 30 m a.s.l.) (control and warm site) along an altitudinal gradient. Three 3 × 3 m plots were located in an open area at each site. Below-ground (0.8 m deep) in each plot was surrounded by concrete brick wall bonding with ceramic tile to prevent the lateral or vertical movement of water or element from the surrounding soils. There was one hole at the top and the bottom of the wall, respectively. In April 2012, soil and individual seedlings that was 1-year-old were collected from a coniferous and broadleaved mixed forest that near the control site. Three different layers of soils (0–20, 20–40 and 40–70 cm) were homogenized separately. Seedlings were stored in shade containers with soil from the collection sites. In May 2012, three different layers of soils were transferred into the plots correspondingly. The seedlings were transplanted into the plots in a randomized block design (n = 6 replicates per species).

The six species included in this study were specifically selected due to their common occurrence and distribution range (existence in almost all regions along the altitudinal gradient) from mixed forest. They included *Schima superba* Gardn. et Champ*, Syzygium rehderianum* Merr. et Perry*, Machilus breviflora* (Benth.) Hemsl*, Pinus massoniana* Lamb.*, Castanopsis hystrix* Hook. f. et Thomson ex A. DC*, Ardisia punctata* Lindl. All species were evergreen, ensuring that their leaves are exposed to the full seasonal changes of temperature. Coniferous and broadleaved species (*P*. *massoniana* vs other species) were chosen.

### Environmental monitoring

A meteorological observation was installed in each experimental plot to mointor air temperature (HMP155A, Vaisala, Finland), soil profile temperatures (CT 109, Campbell, USA) and volumetric soil water content (CS616, Campbell, USA) at 10 cm depth. Each meteorological observation was connected with a data loggers (CR1000, Campbell, USA) to record data every 10 minutes since May 2013.

### Gas exchange measurements

Photosynthetic seasonal patterns were measured on three newly developed and fully expanded leaves per species in each plot. Leaf net photosynthesis (*A*_sat_) was measured using a portable open path gas exchange system (Li-6400; Li-Cor, Inc., Lin-coln, NE, USA) with red-blue light source (6400-02B). The photosynthetic photon flux density was maintained at 1200 μmol m^−2^s^−1^ and [CO_2_] was maintained at 400 μmol m^−2^s^−1^.

On 25–26 October 2013, *A*_sat_ to intercellular CO_2_ (*A*_sat_-*C*_i_ curves) were measured at PPFD of 1200 μmol m^−2^s^−1^, midday growth temperature (22 °C or 23 °C), relative humidity of 50–60% and leaf-to-air VPD between 1.0 and 2.0 kPa, by raising cuvette CO_2_ in 9 steps (0, 50, 100, 200, 300, 400, 600, 800 and 1800 μmol mol^−1^). Analysis of *A*_sat_*–Ci* response curves involved calculation of parameters potentially limiting to photosynthesis: *V*_cmax_ (maximum rate of photosynthetic carboxylation, μmol m^−2^s^−1^), *J*_max_ (maximum rate of photosynthetic electron transport, μmol m^−2^s^−1^) and TPU (triose-phosphate utilization, μmol m^−2^s^−1^). This was achieved using Photosynthesis Assistant (v1.1, Dundee Scientific, Dundee, UK) which uses a biochemical model describing *A*_sat_^9^.

### Data analysis

Repeated-measures ANOVA was used to investigate the effects of translocation on environmental factors, seasonal *A*_sat_ and *g*_s_ in each tree species over the experimental time. The differences in *V*_cmax_, *J*_max_, TPU between the control and warm sites were analyzed by t-test. As there were differences in *A*_sat_ and *g*_s_ between the six tree species, an effect-size index, relative effect (RE)^57^,^58^, was used to estimate the responses of *A*_sat_ to translocation in the six tree species. RE is quantified by the ratio of the variable in the experimental group to the control group minus one. Relationships between *A*_sat_, *g*_s_ and VPD were analyzed using linear regression analysis. Results were considered significant in all cased if *p* < 0.05. All analyses were performed in SPSS (SPSS 17.0 for windows, USA).

## Additional Information

**How to cite this article**: Li, Y. *et al.* Warming effects on photosynthesis of subtropical tree species: a translocation experiment along an altitudinal gradient. *Sci. Rep.* **6**, 24895; doi: 10.1038/srep24895 (2016).

## Figures and Tables

**Table 1:** Repeated-measures ANOVA of translocation, measuring time and their interactions on net photosynthetic rate (*A*_sat_) and stomatal conductance (*g*_s_).

Effect	*Schima superba*	*Castanopsis hystrix*	*Syzygium rehderianum*	*Pinus massoniana*	*Machilus breviflora*	*Ardisia lindleyana*
A_sat_						
Translocation	8.241**	3.224	2.655	42.091***	4.338*	22.103***
Time	15.554**	7.132***	9.749***	27.172***	5.807***	7.742***
Translocation*Time	3.299**	1.094	3.324**	10.790***	1.552	1.325
g_s_						
Translocation	3.694	0.636	7.956**	10.731**	2.539	24.337***
Time	17.314***	9.112***	18.092***	56.615***	19.352***	11.784***
Translocation*Time	2.471^*^	2.785**	4.848***	7.617***	2.368*	3.655***

^*^Statistically significant at *p* < 0.05.

^**^Statistically significant at *p* < 0.01.

^***^Statistically significant at *p* < 0.001.

**Table 2:** Summary of net photosynthetic rate (*A*_sat_) and stomatal conductance (*g*_s_) at saturating light for species growth in the warm and control site.

Species	Site	*A*_sat_ (μmol m^−2^s^−1^)			*g*_s_ (mol m^−2^s^−1^)		
		Year	Wet season	Dry season	Year	Wet season	Dry season
*Schima superba*	Control site	12.34 ± 3.11	12.84 ± 1.24	12.31 ± 1.18	0.211 ± 0.005	0.254 ± 0.021	0.182 ± 0.003
	Warm site	13.26 ± 2.62	14.39 ± 0.75	11.90 ± 0.49	0.232 ± 0.006	0.315 ± 0.022	0.193 ± 0.004
*Castanopsis hystrix*	Control site	7.75 ± 0.73	8.75 ± 1.04	7.03 ± 0.98	0.163 ± 0.004	0.219 ± 0.008	0.120 ± 0.010
	Warm site	7.00 ± 0.62	8.68 ± 0.62	5.79 ± 0.67	0.171 ± 0.005	0.285 ± 0.010	0.104 ± 0.012
*Syzygium rehderianum*	Control site	6.10 ± 0.52	6.65 ± 0.82	5.58 ± 0.71	0.097 ± 0.013	0.130 ± 0.017	0.075 ± 0.007
	Warm site	6.78 ± 0.81	9.13 ± 0.87	5.10 ± 0.60	0.133 ± 0.013	0.224 ± 0.014	0.066 ± 0.008
*Pinus massoniana*	Control site	8.13 ± 0.80	8.92 ± 1.53	7.67 ± 0.84	0.201 ± 0.008	0.297 ± 0.021	0.133 ± 0.005
	Warm site	9.69 ± 0.87	8.97 ± 1.47	9.93 ± 1.14	0.248 ± 0.010	0.362 ± 0.025	0.167 ± 0.006
*Machilus breviflora*	Control site	6.46 ± 0.88	8.20 ± 1.45	5.22 ± 0.91	0.124 ± 0.004	0.195 ± 0.015	0.074 ± 0.008
	Warm site	7.23 ± 0.65	8.10 ± 1.15	6.61 ± 0.75	0.135 ± 0.004	0.209 ± 0.012	0.081 ± 0.008
*Ardisia lindleyana*	Control site	6.10 ± 0.42	7.12 ± 0.60	5.37 ± 0.41	0.113 ± 0.007	0.153 ± 0.011	0.084 ± 0.006
	Warm site	7.77 ± 0.62	9.60 ± 0.56	6.45 ± 0.62	0.182 ± 0.008	0.289 ± 0.014	0.105 ± 0.007

Data are means ± SE.
